# The Link between Occurrence of Class I Integron and Acquired Aminoglycoside Resistance in Clinical MRSA Isolates

**DOI:** 10.3390/antibiotics10050488

**Published:** 2021-04-23

**Authors:** Ahmed M. El-Baz, Galal Yahya, Basem Mansour, Mohamed M. A. El-Sokkary, Reem Alshaman, Abdullah Alattar, Amira M. El-Ganiny

**Affiliations:** 1 Microbiology and Biotechnology Department, Faculty of Pharmacy, Delta University for Science and Technology, Gamasa 11152, Egypt; elbaz_pharmacy@yahoo.com; 2 Microbiology and Immunology Department, Faculty of Pharmacy, Zagazig University, Zagazig 44519, Egypt; galalmetwally2020@gmail.com; 3 Department of Molecular Genetics, TU Kaiserlautern, 67663 Kaiserslautern, Germany; 4 Pharmaceutical Chemistry Department, Faculty of Pharmacy, Delta University for Science and Technology, Gamasa 11152, Egypt; basem2412@yahoo.com; 5 Microbiology and Immunology Department, Faculty of Pharmacy, Mansoura University, Mansoura 35516, Egypt; m_elsokkary2022@yahoo.com; 6 Department of Pharmacology and Toxicology, Faculty of Pharmacy, University of Tabuk, Tabuk 471, Saudi Arabia; ralshaman@ut.edu.sa (R.A.); aalattar@ut.edu.sa (A.A.)

**Keywords:** MRSA, integron I, aminoglycoside resistance, sequencing, *aadA* gene variants, *aacA4* gene

## Abstract

Methicillin-resistant *Staphylococcus aureus* (MRSA) is a major cause of nosocomial infections because of its high resistance. Here, we study the antibiotic resistance in MRSA clinical isolates and their relation to integron I occurrence. A total of 88 clinical *Staphylococcus* *aureus* isolates were collected. MRSA were identified by the disk diffusion method (DDM) and confirmed by PCR, and antibiogram was determined by DDM. Integron I, II and the *aacA4* gene were investigated by PCR. Integrase-positive strains were analyzed for the presence of resistance gene cassettes by sequencing. All isolates were identified as MRSA by DDM and confirmed by PCR. All isolates were resistant to ampicillin and cefoxitin. Concerning aminoglycosides, the frequency of resistance was reported for streptomycin (60.7%), tobramycin (37.1%) gentamicin (36%), and for amikacin (15.9%). Integron I was detected in 41 isolates (46.6%), while integron II was detected in three isolates (3.4%). Sequencing of the integron I-cassette indicated the exclusive prevalence of *addA* gene variants mediating aminoglycoside resistance. The *aacA4* gene was found in DNA of 31 isolates (35.22%). This study revealed the high existence of MRSA. Furthermore, the *AacA4* gene and class I integron harboring *aadA* gene were predominant in MRSA isolates.

## 1. Introduction

*Staphylococcus aureus* is a major human pathogen responsible for a wide range of infections including superficial and fatal systemic infections [1]. *S. aureus* is identified as one of the world’s leading causes of nosocomial infections [2]. In the past, infections caused by *S. aureus* were well-controlled by penicillin [3]. With the misuse of penicillin, penicillin-resistant *S. aureus* and methicillin-resistant *S. aureus* (MRSA) have appeared; MRSA resistance is mediated by the *mecA* gene [4,5]. MRSA has rapidly become the most frequently occurring resistant pathogen that leads to high rates of morbidity and mortality [6,7]. In the last few decades, multidrug resistant (MDR)-MRSA isolates have become prominent in Egypt due to several factors including carriage between healthcare workers, persistence in the hospital environment and the misuse of antibiotics [8,9].

Several mechanisms involving mobile genetic elements, such as plasmids, transposons and integrons, have been shown to contribute to the spread of antibiotic resistant genes [10]. Integrons are mobile-DNA elements that play an important role in the spread of antibiotics and biocides resistance among bacteria [11,12]. Integrons I and II are the classes of integrons that are common in Gram-positive bacteria, including *S. aureus* [13,14].

A complete functional integron consists of three elements: the integrase gene (*intI*) encoding an integrase protein, a recombination site (*attI)* and a promoter (Pc) gene that directs transcription of the gene cassettes. The integrase protein catalyzes recombination between incoming gene cassettes and the recombination site [15]. Integrons usually carry antibiotic resistance genes that contribute to resistance against aminoglycosides, macrolides, β-lactams, chloramphenicol, and sulfonamides [16].

Aminoglycosides are bactericidal agents that play an important role in the anti-staphylococcal therapies. They act by binding specifically to 16S rRNA of the 30S ribosomal subunit, thus interfering with protein synthesis [17]. Gentamicin, tobramycin and amikacin are the most active aminoglycosides against staphylococci and are often used in combination with other groups such as β-lactams [18]. The main mechanism of aminoglycoside resistance is drug inactivation by aminoglycoside-modifying enzymes (AMEs) that decrease aminoglycosides’ affinity to their natural target (16S rRNA).

The most important AMEs are adenylyltransferase (ANT), acetyltransferase (AAC), and phosphotransferase (APH) enzymes [19]. The AACs acetylate an amino group, while the ANTs and APHs adenylate and phosphorylate a hydroxyl group of the antibiotic, respectively. Some AACs are found as fusion proteins located adjacent to other AMEs such as APH and ANT [17,20].

**Figure 1:**
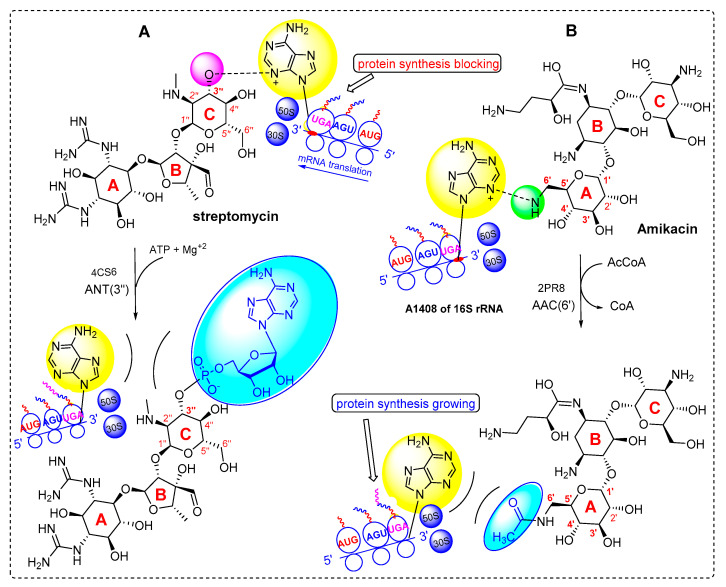
Mechanism of action of AMEs, (**A**) the adenyltransferase *aadA* (PDB; 4CS6) adenylate streptomycin at 3″ position, (**B**) acetyltransferase AAC (6′)-Ib (PDB; 2PR) acetylate amikacin at the 6′ position.

Four types of adenylyltransferases have been identified, including: ANT (2″), ANT (3″), ANT (4′), and ANT (6′). The ANT (3″) are the most common ANT enzymes, they specify resistance to streptomycin, and their coding genes are commonly named *aadA*; at least 22 highly related *aadA* gene variants are found in GenBank. The *aadA* genes exist as gene cassettes and are part of integrons, plasmids or transposons [17,21]. The ANT (3″) adenylates streptomycin at 3″-position (labelled pink) give rise to the cleavage of drug/16S-rRNA complex, restoring the function of ribosomes (Figure 1A).

The AACs are subdivided into several groups based on the position where the acetyl group is inserted in the acceptor aminoglycoside drug. Known AACs catalyze acetylation at the 1 (AAC (1)), 3 (AAC (3)), 2′ (AAC (2′)), or 6′ (AAC (6′)) positions. AAC (6′) enzymes are the most common group of AACs that contain more than 40 enzymes [22]. A plasmid-mediated acetyltransferase AAC(6′)-Ib, also known as *aacA4*, specify resistance to several aminoglycosides including amikacin [20]. AAC (6′) modifies the aminoglycosides (gentamicin, tobramycin and amikacin) by the acetylation of the amino group at 6’-position (labelled green), setting the 16S-rRNA receptor free and turning the ligand into an inactive drug (Figure 1B).

The main purpose of the current study is to investigate the resistance profile of clinical MRSA isolates, to determine the frequency of class I and II integrons in these isolates, and to investigate the pattern of resistance linked to integron I-positive/cassette-positive isolates especially resistant against aminoglycosides. Furthermore, aminoglycoside resistance due to the *aacA4* gene was investigated.

## 2. Results

### 2.1. Identification of S. aureus Isolates

A total of 88 non-duplicate clinical *S. aureus* isolates originated from wounds (46%), urine (30%), sputum (13%), and blood (11%) were identified in this study. The majority of cases (57.95%) were males, 32.95% of the total number of cases were <19 years old, 37.5% of cases were between 19 to 40 years, 11.36% of cases were between 41 and 60 years, and 18.18% of them were >60 years. All *S. aureus* isolates showed golden yellow colonies on nutrient agar, and yellow fermentation on mannitol salt agar. Microscopically, all isolates were positive in Gram staining with the arrangement in grapes clusters. Biochemically, all isolates were mannitol fermenters. All the isolates were identified phenotypically as MRSA by the disk diffusion method using a cefoxitin disk.

### 2.2. Antimicrobial Susceptibility Testing

**Figure 2:**
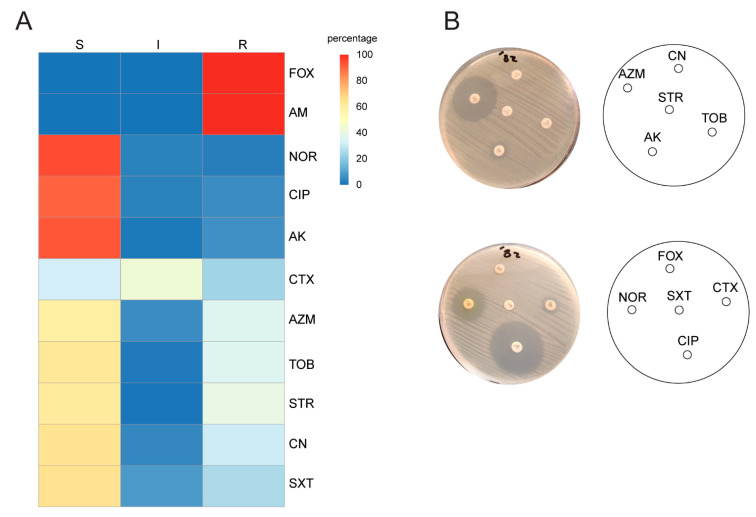
Antimicrobial susceptibility of MRSA isolates: (**A**) representative heatmap depicting the observed antibiogram for the *S. aureus* isolates to different antimicrobial chemotherapeutics. Cefoxitin (FOX), ampicillin (AM), cefotaxime (CTX), ciprofloxacin (CIP), norfloxacin (NOR), amikacin (AK), tobramycin (TOB), gentamicin (CN), streptomycin (STR), azithromycin (AZM), and sulfamethoxazole/trimethoprim (SXT). Sensitive (S), intermediate resistant (I), resistant (R). The heatmaps were created using R version 4.0.1 with the package ggplot2 version 3.3.1 [23]; the heatmap matrix was retrieved from scored susceptibility identified by DDM in (**B**). (**B**) Antimicrobial susceptibility of *S. aureus* isolates against different antibiotics by DDM.

The antimicrobial susceptibility test revealed that all the isolates were resistant to ampicillin and cefoxitin (100%). Frequency of resistance was commonly reported with cefotaxime (67.41%) and streptomycin (60.67%), while intermediate resistance was detected in azithromycin (43.82%), tobramycin (37.1%), gentamicin (35.95%), and sulfamethoxazole/trimethoprim (35.33%). Resistance was rarely observed with amikacin (15.9%), ciprofloxacin (9%), and norfloxacin (5.68%), (Figure 2). Forty-nine of the isolates (55.7%) were multidrug resistant (MDR).

### 2.3. Confirmation of MRSA Identification, Screening of Integron I, II and aacA4 Genes by Polymerase Chain Reaction (PCR)

**Figure 3:**
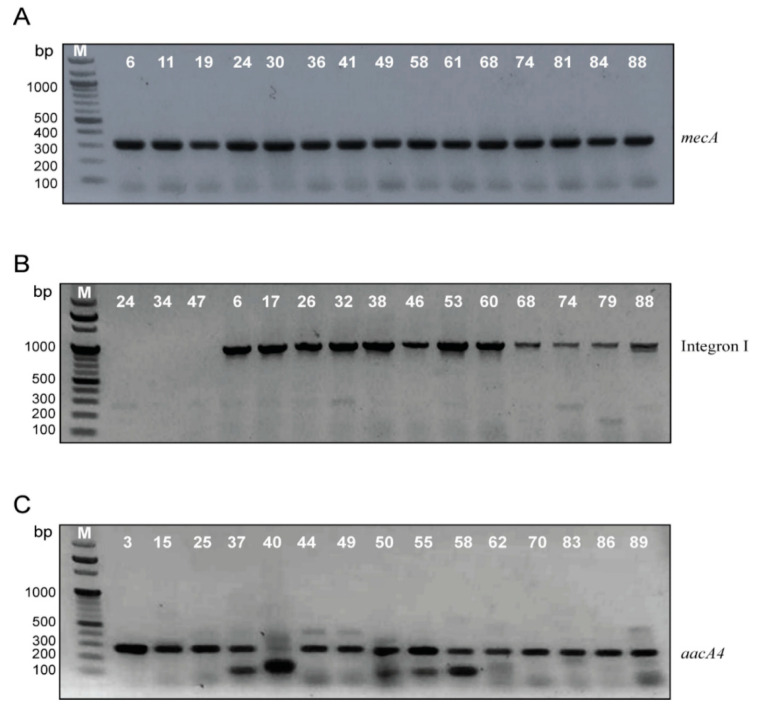
Electrophoretic graph of PCR products on 1.5% agarose gel stained with ethidium bromide for (**A**) *mecA* gene among *S. aureus* isolates, positive isolates gave a single band of 310 bp. (**B**) Integron I amplicon among MRSA, positive isolates gave a single band of either 250 bp or 1000 bp. (**C**) *aacA4* gene among MRSA, positive isolates gave a single band of 200 bp.

PCR was used for the amplification of the *mecA* gene to confirm MRSA identification. All the isolates gave a single band at 310 bp matched to the *mecA* gene (Figure 3A).

PCR was also used for integron detection using a primer specific for class I integron (*intI1*). Integron I was detected in 41 isolates (46.6%) with a variable amplicon size, 35 isolates (39.8%) with a single band of approximately 1000 bp (Figure 3B), and only six isolates (6.8%) produced a single band of 250 bp. Integron II with 200 bp amplicon was detected in only three isolates (3.4%).

The *aacA4* gene encoding for aminoglycoside resistance was detected in DNA of 31 isolates (35.22%) with an amplicon size of 300 bp (Figure 3C)

### 2.4. Sequencing of Integron I-Positive Isolates

**Figure 4:**
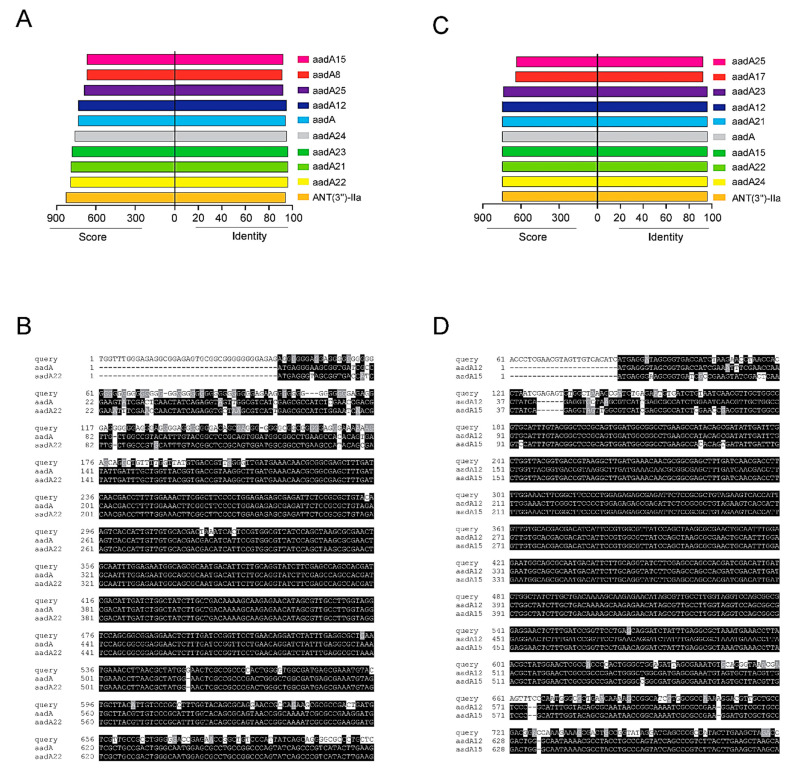
(**A**) Bar blots indicating the identity percentage and covered score of the sequenced query 1 against the most likelihood AMR genes recognized by the Comprehensive Antibiotic Resistance Database (CARD). (**B**) Multiple sequence alignment of one category of the identified AMR genes (query 1) with the sequences of aadA and aadA22 genes retrieved from banked microbial resistome that give a high identity to the queried sequence analyzed via CARD; the available sequences are aligned via ClustalW and BOXSHADE and were employed to highlight the multiple alignment. Black shading indicates that the residue is identical to the column consensus; gray shading indicates that the residue is not identical but at least similar to the column consensus; and no shading indicates that the residue is neither identical nor similar to the consensus [24]. (**C**) Bar blots indicating the identity percentage and covered score of the sequenced query 2 against the most likelihood AMR genes retrieved from CARD. (**D**) CLUSTAL W multiple sequence alignment of the other category of the identified AMR genes (query 2) with the sequences of aadA12 and aadA15 genes retrieved from banked microbial resistomes that give a high identity to the queried sequence using CARD; the available sequences are aligned and shaded via ClustalW and BOXSHADE, as previously described.

Integron-borne resistance was investigated by PCR amplification for the integrase gene followed by sequencing the variable region and 3′-conserved regions. The results are shown in Figure 4. Multiple sequence alignment showed that integron I of 1 kb size has a sequence similarity with *aadA* gene variants (*aadA22*, *aadA12*, and *aadA15*), while integron I with 250 bp does not contain any gene (empty) cassette (Table 1). Unique sequences analyzed by the Comprehensive Antibiotic Resistance Database (CARD) detected two major categories of cassette arrays coding aminoglycosides resistance genes with a high degree of inferred sequence homology. The two frequently detected AMR genes both belonging to the aminoglycoside nucleotidyltransferase are usually responsible for nucleotidylylation of streptomycin at the hydroxyl group at position 3″(ANT(3″)). One of the two subfamilies shows high identity (95 to 98%) to *aadA15*, identified in the resistome of *Pseudomonas aeruginosa* and *aadA12*, identified in the resistome of *Escherichia coli*, *Yersinia enterocolitica* and *Salmonella enterica.* The other subfamily shows a 95% identity to *aadA*, the aminoglycoside nucleotidyltransferase gene encoded by plasmids, transposons, and integrons in *Enterobacteriaceae*, *A. baumannii*, *P. aeruginosa* and *Vibrio cholerae*, and a 95% identity to *aadA22* in *S. enterica* and *E. coli.* Other closely related resistomes with sequence variants for the two AMR gene variants were detected by the sequence recognition analysis against a library of identified aminoglycosides resistomes annotated in the resistome database (Figure 4A,C).

### 2.5. Classification of Isolates According to Resistance Profile, Integron I and aacA4 Presence

Merging the results retrieved from the molecular analysis of the most probable elements for resistance, the integron I-positive isolates can be divided into three groups. Group one includes 13 isolates which are integron I-positive (cassette-positive) and *aacA4*-positive; all of these isolates were resistant to all aminoglycosides including amikacin. Group two includes 22 isolates that are integron I-positive (cassette-positive) and *aacA4*-negative; these isolates were resistant to streptomycin only, while Group three includes six isolates only integron-positive (cassette-negative) and *aacA4*-negative; these isolates are sensitive to all aminoglycosides as shown in Table 1.

Table 2 contain the characters of the integron I-negative, *aacA4*-positive isolates (18 isolates). A total of 17 of these isolates were resistant to all aminoglycosides except amikacin, while only one isolate was resistant to all aminoglycosides including amikacin.

## 3. Discussion

The screening of antibiotic resistance in pathogenic bacteria, especially *S. aureus*, is very important for infection control practice. MRSA has been labelled as a ‘super bug’ due to its widespread resistance to commonly used antibiotics [25]. The present study was performed to investigate the resistance of MRSA isolates from Egyptian hospitals and their relation to integron I occurrence.

Phenotypic MRSA identification was confirmed by the disk diffusion method using a cefoxitin disk; all of the isolates were resistant to cefoxitin (100%). The usefulness of the cefoxitin disk in predicting methicillin resistance has been reported, with sensitivity values reaching 100% [26,27]. However, identification of the *mecA* gene is the most reliable method of detecting MRSA isolates [28]. Hence, *mecA* amplification by PCR was performed to confirm MRSA identification. All of the isolates were *mecA*-positive, and a high prevalence (75%) of MRSA was reported in Nepal [29] and 72% in Eritrea [30], while lower rates were reported in Europe, ranging from 0.9% in the Netherlands to 56% in Romania [6]. The observed existence of MRSA in this study may be related to several factors, including: carriage between healthcare workers, persistence in the hospital environment and the misuse of antibiotics [9,31].

The rise of MDR *S. aureus* strains, particularly MRSA, is a serious problem in the treatment and control of staphylococcal infections [32]. Approximately 55% of the MRSA isolates were MDR. A quite similar percentage of MDR (47%) was reported recently in *S. aureus* isolates from dairy cattle in China [33], while a higher percentage of MDR (78%) was reported among clinical *S. aureus* isolates in Iran [13].

All of the isolates in this study were resistant to ampicillin and cefoxitin (100%). Similarly, Gurung and his colleagues reported that all MRSA isolates were resistant to penicillin and cefoxitin [29]. Regarding aminoglycoside resistance, resistance was often reported with streptomycin (60.67%), intermediate resistance with tobramycin (37.1%), and gentamicin (35.95%), while resistance was less frequently observed for amikacin (15.9%). However, slightly lower resistance to gentamicin (10.2%) was previously reported by Gurung and his colleagues [29]. The low resistance detected for ciprofloxacin (9%), and norfloxacin (5.68%), makes these drugs the best treatment options for MRSA infections in our hospitals.

The occurrence of integrons in clinical isolates and their role in antimicrobial resistance have been widely studied in the past few decades [11,12,34]. In the current study, integron I (with variable amplicon) was detected in 41 isolates (46.6%), while integron II (with 200 bp amplicon) was detected in only three isolates (3.4%). Similarly, class I integron was found in 42.5% of nosocomial MRSA isolates (76/179) in China [25]. Furthermore, another study reported that the rate of detection of integron I (72.6%) was more than integron II (35.2%), but with higher percentages than our study [13]. In contrast, a recent study in Iran reported a lower prevalence of class I integron (24.8%), and complete absence of class II in their isolates at all [16].

Cassette genes encoding resistance to aminoglycosides were found to be predominant in the class I integron. The *aadB* and *aadA* genes encoding aminoglycoside resistance were most commonly found. *aadB* confer resistance to gentamicin, tobramycin, and kanamycin, while *aadA* confer resistance to streptomycin [13]. In this study, integron I cassette gene sequencing revealed the presence of different alleles of the *aadA* gene (aadA12, 15, 22, 23) in 35 isolates (39.8%). Similarly, the *aadA* gene was frequently detected in MRSA isolates harboring class I integron from China [25]. In fact, the *aadA* gene cassettes were found to be the most often reported cassettes in different bacterial isolates harboring class I integron [35]. Furthermore, similar allelic diversity of the *aadA* gene has been reported among Gram-negative isolates, including clinical *Klebsiella pneumoniae* strains [36,37].

In the current study, only six of the integron I-positive isolates with 250 bp amplicon (6.8%) were found to have empty cassette. Similarly, it was previously reported by Li and Zhao) (2018) in China [33] that the class I integron with a 153 bp size does not usually harbor any gene cassette, but this empty integron was found in 21.5% of their isolates.

Previous studies reported the occurrence of the *aacA4* gene among integron cassettes but with lower existence than *aadA* genes [13,33]. However, none of the current isolates contain *aacA4* gene within integron I; however, the *aacA4* gene was detected in the genomic DNA of 31 isolates. In our study, 13 of the *aacA4*-positive isolates were also integron I/cassette-positive (carrying the *aadA* gene variant); those isolates were resistant to all tested aminoglycosides including amikacin. Other isolates that are integron I-positive (carrying the *aadA* gene) but negative for the *aacA4* gene, were resistant to streptomycin only. The results obtained are in consistence with the literature stating that *aadA* variants confer resistance to streptomycin and spectinomycin only [38,39].

**Figure 5:**
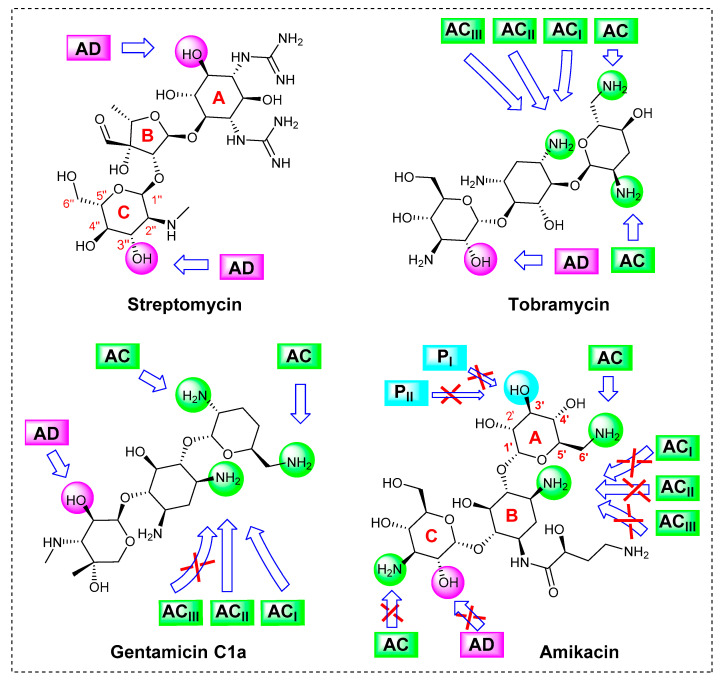
Chemical structures of aminoglycosides under investigation showing positions targeted by modifying enzymes: acetyltransferase (AC), adenylyltransferase (AD) and phosphotransferase (P). Gentamicin and amikacin show resistance to some AMEs. Adapted from [42] with some modifications.

Almost all the integron I-negative but *aacA4*-positive isolates (17/18) were resistant to all aminoglycoside except amikacin. Overall, it seems that the presence of both adenylyltransferase (*aadA*) and acetyltransferase (*aacA4*) is essential for amikacin resistance. Previous studies related amikacin resistance in *E. coli* to the presence of *aacA4* and *aacA7* together [40]; along the same lines with our finding, it was reported that the ANT(3″) enzymes confer more resistance against amikacin in *Serratia marcescens* by increasing the affinity of the aminoglycoside to bind AAC(6′) enzyme [41]. Only one isolate (1/18) was integron I-negative and *aacA4*-positive, and it showed resistance to amikacin; in this isolate, other mechanisms could play a role in such resistance. For example, reduced drug uptake was reported as a mechanism of resistance to amikacin. Interestingly, amikacin is considered the most resistant drug to the action of AMEs, as shown in Figure 5 [20].

Furthermore, integron I-positive isolates showed resistance to other classes of antimicrobials (beta-lactam, macrolide, quinolone, sulphonamides); however, related resistance gene cassettes were not found on integron I, suggesting non-integron sources of resistance to these antimicrobials. Similarly, the absence of any gene cassette conferring resistance to erythromycin, clindamycin, tetracycline, penicillin, and ampicillin was previously reported by Li and Zhao (2018) in China [33] although the isolates were resistant to these antibiotics, which suggest other sources of such resistance by other different mobile DNA elements, such as plasmid and transposon, or other resistance genes under chromosomal control [13,33].

## 4. Materials and Methods

### 4.1. Bacteria Isolation and Identifications

A total of eighty-eight *S. aureus* isolates were collected from clinical laboratories in Mansoura University Hospital, Egypt, from different clinical samples (wounds, urine, sputum, and blood). All isolates were identified using standard microbiological tests, including Gram stain and growth on mannitol salt agar [43].

### 4.2. Antimicrobial Susceptibility

Antimicrobial susceptibility was determined by the disk diffusion method according to the Clinical and Laboratory Standard Institute [44]. Susceptibility testing was performed using antibiotics from 5 classes including: ampicillin (Am, 10 µg), cefoxitin (FOX, 30 µg), cefotaxime (CTX, 30 µg), ciprofloxacin (CIP, 5 µg), norfloxacin (NOR, 10 µg), tobramycin (TOB, 10 µg), gentamicin (CN, 10 µg), streptomycin (STR, 10 µg), amikacin (AK, 30 µg), azithromycin (AZM, 15 µg), and sulfamethoxazole/trimethoprim (SXT, 1.25/23.75 µg). Antibiotic disks were obtained from Oxoid, (Hampshire, England). The results were interpreted according to the criteria indicated in the CLSI guidelines [44].

### 4.3. Amplification of Integron I, Integron II, mecA and aacA4 Genes Using PCR

Total DNAs from different samples were extracted using the boiling method as described previously [45]. Each PCR reaction was performed using a Biometra T-personal thermocycler (Goettingen, Germany), in 25 reaction mixtures containing 2.5 µL of DNA, 12.5 µL of MyTaq™ red mix (Bioline Co., London, UK), 1 µL of forward primer (10 µM), 1 µL of reverse primer (10 µM) and nuclease free water to 25 µL.

For confirmation of MRSA identification, *mecA* genes were detected by polymerase chain reaction (PCR) using the primers listed in Table 3 [46]. the cycling conditions include heating at 94 °C for 10 min, then 30 cycles of 94 °C for 30 s, annealing at 52 °C for 30 s and 72 °C for 45 s and finally heating at 72 °C for 5 min.

Different classes of integrons (I and II) were also detected by PCR using the primers listed in Table 1 [47]. The cycling conditions include heating at 94 °C for 5 min, then 35 cycles of 94 °C for 30 s, 49 °C and 52 °C for 20 s and 72 °C for 30 s and finally heating at 72 °C for 3 min.

Furthermore, the *aacA4* gene (mediating aminoglycoside resistance) was detected in the total DNA of *S. aureus* isolates by PCR as previously described [48]. The cycling conditions include heating at 94 °C for 5 min, then 35 cycles of 94 °C for 30 s, 54 °C for 45 s and 72 °C for1 min, and then finally heating at 72 °C for 10 min. The PCR products as well as GeneRuler 100 bp plus DNA ladder (Thermo scientific, Waltham, MA, USA) were separated on 1.5% agarose gel, stained with ethidium bromide, and visualized by UV transilluminator.

### 4.4. Sequencing of Amplified Integrons

PCR amplicons comprising integrons were cleaned up using QIAquick PCR Purification (Qiagen, Hilden, Germany) according to the manufacturer’s instructions. DNA was eluted using the elution buffer, and the concentration of eluted DNA was measured using a NanoDrop^TM^ 2000/2000c Spectrophotometer (Thermo Fisher Scientific, Waltham, MA, USA) including the quantification of 260/280 and 260/230 nm ratios with 1 µL of sample.

Purified PCR samples were used for sequencing according to service requirements; 5 µL of template DNA (20–80 ng) were mixed with 5 µL of the specific primers (5 pmol/µL). PCR samples were sequenced using the Illumina HiSeq platform using 300 PE chemistry (GATC-Biotech, Konstanz, Germany, now part of Eurofins Genomics Germany GmbH).

Multiple sequence alignment of the identified AMR genes (query 1 and 2) with the sequences of the *aadA* and *aadA22* genes retrieved from banked microbial resistomes that give high identity to the queried sequence were analyzed via the Comprehensive Antibiotic Resistance Database (CARD) (https://card.mcmaster.ca/analyze/blast), access date 15 January 2021. The available sequences aligned via ClustalW and BOXSHADE were employed to highlight the multiple alignment.

## 5. Conclusions

The current study revealed the alarming existence of MRSA isolates. Notable levels of resistance to penicillin, cephalosporins and aminoglycosides were observed. Both the *AacA4* gene and class I integrons harboring the *aadA* gene (mediating resistance to aminoglycosides) were predominant in MRSA isolates. The link between integrons and aminoglycoside resistance in MRSA isolates is notable and could be clinically important.

## Figures and Tables

**Table 1:** Resistance profile and characters of integron I-positive MRSA isolates.

Isolate No	Integron I (bp)	Sequence Profile of Integron I	*aacA4* Gene	Antibiotic Resistance Profile
6	1000	query 2 (*aadA*12,15)	−	FOX, AM, CTX (I), STR, SXT
8	1000	query 1 (*aadA*22,23)	−	FOX, AM, STR, CTX(I), SXT(I)
11	1000	query 1 (*aadA*22,23)	−	FOX, AM, CTX (I), STR
13	1000	query 1 (*aadA*22,23)	−	FOX, AM, CTX, STR AZM,
15	1000	query 2 (*aadA*12,15)	+	FOX, AM, CTX(I), STR, TOB (I), CN (I), AK(I) AZM
17	1000	query 1 (*aadA*22,23)	−	FOX, AM, CTX, STR, SXT(I), AZM
19	1000	query 1 (*aadA*22,23)	−	FOX, AM, CTX, STR, SXT
20	1000	query 1 (*aadA*22,23)	−	FOX, AM, CTX(I), STR, SXT
21	1000	query 1 (*aadA*22,23)	+	FOX, AM, CTX(I), STR, CN, TOB, AK, AZM
24	250	Empty cassette	−	FOX, AM
25	1000	query 2 (*aadA*12,15)	+	FOX, AM, CTX(I), STR, CN, TOB, AK, AZM(I)
26	1000	query 1 (*aadA*22,23)	−	FOX, AM, CTX, STR, SXT
27	1000	query 1 (*aadA*22,23)	−	FOX, AM, CTX (I), STR, AZM
28	1000	query 2 (*aadA*12,15)	−	FOX, AM, STR
29	250	Empty cassette	−	FOX, AM
30	1000	query 1 (*aadA*22,23)	−	FOX, AM, STR
31	250	Empty cassette	−	FOX, AM
32	1000	query 2 (*aadA*12,15)	−	FOX, AM, CTX (I), STR, SXT
34	250	Empty cassette	−	FOX, AM
35	1000	query 1 (*aadA*22,23)	−	FOX, AM, STR, SXT
36	1000	query 1 (*aadA*22,23)	−	FOX, AM, STR, AZM
37	1000	query 2 (*aadA*12,15)	+	FOX, AM, STR, AK, CN, TOB, AZM, SXT
38	1000	query 1 (*aadA*22,23)	−	FOX, AM, STR, AZM
41	250	Empty cassette	−	FOX, AM
42	1000	query 1 (*aadA*22,23)	+	FOX, AM, CTX (I), STR, AK, CN, TOB, AZM, SXT
46	1000	query 2 (*aadA*12,15)	+	FOX, AM, CTX (I), STR, AK, CN, TOB, AZM
47	250	Empty cassette	−	FOX, AM
49	1000	query 2 (*aadA*12,15)	+	FOX, AM, CTX, STR, AK, CN, TOB, NOR, CIP, SXT, AZM
53	1000	query 1 (*aadA*22,23)	−	FOX, AM, CTX, STR, AZM
58	1000	query 1 (*aadA*22,23)	+	FOX, AM, CTX(I), STR, CN, TOB, AK, AZM
60	1000	query 1 (*aadA*22,23)	−	FOX, AM, CTX(I), CIP, NOR(I), STR, SXT(I)
61	1000	query 2 (*aadA*12,15)	+	FOX, AM, CTX(I), STR, CN, TOB, AK(I)
65	1000	query 1 (*aadA*22,23)	−	FOX, AM, CTX, STR, AZM
68	1000	query 1 (*aadA*22,23)	−	FOX, AM, CTX, STR, SXT
71	1000	query 2 (*aadA*12,15)	+	FOX, AM, CTX, STR, CN, TOB, AK(I), AZM
74	1000	query 1 (*aadA*22,23)	−	FOX, AM, CTX, STR, SXT
78	1000	query 2 (*aadA*12,15)	−	FOX, AM, CTX(I), STR, SXT
79	1000	query 1 (*aadA*22,23)	−	FOX, AM, STR, SXT
81	1000	query 1 (*aadA*22,23)	+	FOX, AM, STR, AK, CN, TOB, AZM, SXT
84	1000	query 1 (*aadA*22,23)	+	FOX, AM, CTX(I), STR, AK, CN, TOB, AZM, SXT
88	1000	query 2 (*aadA*12,15)	+	FOX, AM, CTX, STR, CN, TOB, AK, AZM

Cefoxitin (FOX), ampicillin (Am), cefotaxime (CTX), ciprofloxacin (CIP), norfloxacin (NOR), amikacin (AK), tobramycin (TOB), gentamicin (CN), streptomycin (STR), azithromycin (AZM), and sulfamethoxazole/trimethoprim (SXT).

**Table 2:** Resistance profile of integron-negative /*aacA4*-positive MRSA isolates.

Isolate No.	Integron I	*aacA4* Gene	Antibiotic Resistance Profile
3	−	+	FOX, AM, CTX (I), CN, TOB, STR,
4	−	+	FOX, AM, CTX, CN (I), TOB, STR, SXT
5	−	+	FOX, AM, CTX (I), CN, TOB, STR, AZM
23	−	+	FOX, AM, CTX, CN, TOB, STR, AZM
40	−	+	FOX, AM, CTX (I), CN, TOB, STR, AZM (I)
43	−	+	FOX, AM, CN, TOB, STR, AZM (I)
44	−	+	FOX, AM, CTX, CIP (I), CN, TOB, STR, AZM
48	−	+	FOX, AM, CTX (I), CN (I), TOB, STR, AZM
50	−	+	FOX, AM, CTX, CN, TOB, STR, AZM
51	−	+	FOX, AM, CTX (I), CN, TOB, STR, AZM
55	−	+	FOX, AM, CTX (I), CN, TOB, STR, AZM
57	−	+	FOX, AM, CTX (I), CN, TOB, STR, AZM
62	−	+	FOX, AM, CTX, CN (I), TOB, STR, SXT
70	−	+	FOX, AM, CTX (I), CN, TOB, STR, AZM
85	−	+	FOX, AM, CN, TOB, STR, AZM (I)
86	−	+	FOX, AM, CTX, CIP (I), CN, TOB, STR, AZM
87	−	+	FOX, AM, CTX(I), CIP, NOR, AK, CN, TOB, STR, AZM, SXT
89	−	+	FOX, AM, CTX (I), CN, TOB, STR, AZM

Cefoxitin (FOX), ampicillin (Am), cefotaxime (CTX), ciprofloxacin (CIP), norfloxacin (NOR), amikacin (AK), tobramycin (TOB), gentamicin (CN), streptomycin (STR), azithromycin (AZM), and sulfamethoxazole/trimethoprim (SXT).

**Table 3:** Primers and amplicon size for *mecA* gene, integron class I, class II and *aacA4* genes.

Primer	Sequence (5′-3′)	Amplicon Size	Target	Annealing Temperature	Reference
*mecA*-F	GTAGAAATGACTGAACGTCCGATAA	310 bp	*mecA* gene	52 °C	[46]
*mecA*-R	CCAATTCCACATTGTTCGGTCTAA				
5′-CS	GGCATACAAGCAGCAAGC	Variable	Intg I gene cassette(s)	49 °C	[47]
3′-CS	AAGCAGACTTGACCTGAT				
Ti-F	ACCTTTTTGTCGCATATCCGTG	Variable	Intg II gene cassette(s)	52 °C	
TI-B	CTAACGCTTGAGTTAAGCC				
*aacA4*F	GCTCTTGGAAGCGGGGACGG	300 bp	*aacA4* gene	54 °C	[48]
*aacA4*R	TCGCTCGAATGCCTGGCGTG				

## Data Availability

The data presented in this study are available on request from the corresponding author.
